# Growth Performance and Carcass Quality of Crossbred Holstein × Beef Heifers and Steers Fed High-Solid Milk in the Preweaning Period

**DOI:** 10.3390/ani16182955

**Published:** 2026-09-20

**Authors:** Evaristo Carrillo-Moreno, Dalia I. Carrillo-Moreno, Evaristo Carrillo-Castellanos, Elizabeth Pérez-Rebolloso, Miguel Mellado

**Affiliations:** 1 Department of Veterinary Medicine, Autonomous Agrarian University Antonio Narro, Torreon 27054, Mexico; evaristocarrillomoreno@outlook.com (E.C.-M.); dalia.ivettecm@gmail.com (D.I.C.-M.); elizabethperezrebolloso@hotmail.com (E.P.-R.); 2 Technological Institute of Torreon, Torreon 27170, Mexico; 3 Department of Animal Nutrition, Autonomous Agrarian University Antonio Narro, Saltillo 25315, Mexico

**Keywords:** beef-on-dairy, early-nutrition, beef grading

## Abstract

This study aimed to determine whether greater weight gains in crossbred calves fed whole milk adjusted to 14% total solids during the preweaning phase persist into the postweaning and finishing phases and influence carcass grading. Eighty Holstein × beef calves (male and female) were randomly assigned to receive either pasteurized whole milk with 11% total solids (11%TS) or pasteurized whole milk supplemented with milk replacer to achieve 14% total solids (14%TS). Calf nutrition was modified only during the preweaning phase (<60 d). Calves fed the 14%TS diet received more nutrients than those fed the 11%TS diet, resulting in greater ADG (971 ± 16 vs. 851 ± 29 g; *p* < 0.01) and weaning weight (98.0 ± 1.29 vs. 93.2 ± 1.85 kg; *p* < 0.05). After weaning, and over a 49-week period, the gap in cumulative ADG in favor of the 14%TS group (+120 g) gradually decreased over time (only −5.6 g per month), yet consistently remained higher than that of the 11%TS group, resulting in a greater (*p* < 0.05) final BW (+17.5 kg) for calves in the 14%TS group than the control group (520.4 ± 3.6 vs. 502.9 ± 7.4 kg). In addition, a greater (*p* < 0.05) proportion of animals was assigned to higher-quality lots (sale lot assignment; 30/40; 75% in lot type 1 for 14%TS vs. 20/40; 50% for 11%TS) and a greater numerical proportion of carcasses was classified as Choice (27/40; 67.5% for 14%TS vs. 20/40: 50% for 11%TS), as determined by USDA-approved automatic grading. Ultimately, these advantages translated into greater estimated revenue per animal (USD 117 per calf) for the 14%TS group than the 11%TS group under USDA beef-grading scenario. In conclusion, the results of this study demonstrate that providing enhanced nutrition to beef-on-dairy calves improves growth performance during the preweaning phase, compared with the control group. This improvement persists over time, reflecting in favorable slaughter outcomes and numerical responses in carcass grading, thereby increasing revenue per animal and reinforcing the strategic value of beef-on-dairy systems. In addition, the financial advantages of implementing a payment method based on USDA beef grading classification are highlighted.

## Simple Summary

Providing enhanced nutrition to Holstein × beef calves during the preweaning stage improves growth performance, reflected in greater average daily gain and weaning weights. The results demonstrate that early nutrition has long-term effects, with the growth advantage established during the preweaning period persisting into the finishing phase. Enhanced nutrition was associated with favorable numerical responses in carcass traits, including heavier carcasses and larger ribeye area, as well as a greater numerical proportion of carcasses classified as Choice. These responses were associated with greater carcass market value and revenue per animal. These findings emphasize the strategic importance of nutrition management in beef-on-dairy systems and its potential practical implications for commercial production.

## 1. Introduction

Beef production based on crossbred dairy cattle has expanded in the United States and Europe [1,2], offering diversification opportunities for dairy farmers. This practice supplies bulls and heifers to cattle feeders and even generates new feeder and finishing business models within farms. Different reproductive strategies have been adopted to incorporate beef semen into dairy operations. Initially, sexed semen was used to improve progeny milk yield, freeing some cows to be inseminated with beef bull semen [3]. More recently, genomic testing has enabled precise selection of cows for sexed semen, maximizing genetic progress [4] while the remaining cows receive beef semen to produce beef × dairy crossbred calves of higher value than straightbred dairy calves [5]. In U.S. herds applying combined strategies (conventional, sexed, and beef semen), 60% of females were genotyped, with calving’s distributed as 19% conventional, 40% sexed dairy, 40% conventional beef, and 1% sexed beef semen [2]. These strategies allow farmers to produce high-quality beef crossbred animals while maintaining replacement heifer inventories and improving herd genetics [4]. Economic models confirm a profitability advantage when implementing such strategies [4,6]. Thus, beef production from crossbred dairy calves should be considered a strategic cash-flow source rather than a byproduct [1].

As dairy–beef calf production increases in Mexico, Ireland, Germany, Switzerland and the United States [1,2,4,7], opportunities arise to improve the preweaning development through nutritional and management strategies. Increasing milk volume or total solid content has been shown to enhance weight gain and feed efficiency during the preweaning period [8,9,10]. Enhanced early-life nutrition has been shown to confer immediate and long-term benefits, including superior growth rates, carcass quality, and reproductive development [11,12]. While studies have focused on the effect of improved nutrition early in life on subsequent reproductive performance [13,14], milk yield in cattle [14,15,16] and productive life [14,17], the long-term consequences of early postnatal nutritional management on growth rate and development in postweaning stage cattle remain less understood [18].

Despite the relevance of these findings for dairy cattle, few studies have evaluated preweaning practices in beef-on-dairy crossbred calves and their long-term effects on postweaning growth and carcass characteristics. Consequently, it remains unclear whether the growth advantage associated with enhanced preweaning nutrition persists after weaning and is reflected in slaughter performance. It was hypothesized that greater preweaning growth rate achieved through enhanced early nutrition would improve final growth rate and subsequently influence carcass characteristics. Accordingly, this study aimed to evaluate the postweaning effects of enhanced preweaning nutrition achieved by increasing milk total solids from 11% to 14% on subsequent growth performance and carcass characteristics of beef-on-dairy calves. An additional objective was to evaluate the economic implications of the enhanced preweaning growth on the subsequent growth performance and carcass characteristics of crossbred Holstein × beef cattle.

## 2. Materials and Methods

### 2.1. Preweaning Management

Animal care and management were in compliance with the Guide for the Care and Use of Agricultural Animals in Research and Teaching [19]. The study adhered to all CONSORT 10 guidelines [20], enabling readers to assess the reliability and validity of the trial findings. The experiment was conducted under ethics permit 38111-425203001-2116 at the Autonomous Agrarian University Antonio Narro. Crossbred (Holstein × beef) calves (*n* = 80; 40 males and 40 females; mean birth weight ± SD = 39.7 ± 3.2 kg) born and raised at a large commercial dairy farm in a hot environment (25 °N, elevation 1155 m, mean annual rainfall 228 mm, mean annual temperature 23.8 °C) were enrolled in this study. After parturition, calves were separated from their dams, and their navels were dipped in a commercial disinfectant solution of 7% iodine tincture. Birth weight and pre-weaning assessments were recorded using a digital scale (PLABA-9, Rhino Maquinaria, Mexico City, Mexico) with a capacity of 1 ton and an accuracy of 0.2 kg.

Calves were immediately placed in individual portable outdoor open pens (2.4 m × 1.2 m) with lateral wooden boards and metal roofs. Pens were bedded with sand and harrowed once a week to keep the bedding dry. In addition, the calf area had a shade net as extra protection from the sun.

All calves received 4 L of high-quality pasteurized colostrum (≥50 mg/mL of IgG; °Brix value ≥ 22%; [21] fed orally via nipple bottle feeders within one h of birth. Calves received a second feeding of 4 L of pasteurized colostrum within the next 10 h of birth.

At birth, each calf was randomly allocated to one of the two treatments. Treatments were balanced by sex, with 20 females and 20 males assigned to each treatment. Mean birth weights (mean ± SE) were 39.5 ± 1.19 and 39.4 ± 1.30 kg for heifers and 44.95 ± 1.34 and 40.10 ± 1.45 kg for steers assigned to the 11% and 14% total solids treatments, respectively. Treatment 1 consisted of pasteurized whole milk containing 11% solids (450 L; DM = 49.5 kg). Treatment 2 consisted of pasteurized whole milk adjusted with milk replacer to achieve 14% solids (450 L; DM = 63.0 kg). The total milk volume for both groups was the same: 450 L in 58 days. For these adjustments, a digital refractometer (Misco palm abbe PA203; Solon, OH, USA) was used to measure the total solids in milk with automatic temperature correction, thereby increasing the accuracy of the results. The enhanced liquid diet was prepared by adding a milk replacer containing 22% crude protein, 15% fat and 0.50% crude fiber (Herd Maker, UltraCare Calf Milk Replacer Land O’Lakes, Inc., Shoreview, MN, USA) to the pasteurized milk.

After pasteurization and before feeding, the milk’s total solids were determined using the digital refractometer to calculate the grams of milk replacer required to achieve 14% TS in the mixture. The milk replacer was weighed with a digital scale (Bar-6, Rhino Maquinaria, Mexico City, Mexico; capacity 20 kg, accuracy 2 g), and mixing was performed for 5 min in a stainless-steel tank with a capacity of 600 L. The digital refractometer was calibrated daily before readings, which were taken on pasteurized whole milk and again after adding milk replacer to confirm solids’ content. In addition, samples were regularly sent to the laboratory for validation of refractometer measurements.

The liquid feeding program, applied during both the early stage and the weaning period, involved a gradual increase or decrease in the volume of milk offered to the calves. Both groups (14% and 11% total solids) were fed according to the following volume schedule: 3 L × 2 times, from 2 to 15 days, 4 L × 2 times, from 16 to 30 days, 5 L × 2 times, from 31 to 53 days, 2 L × 2 times, from 54 to 56 days, 1 L × 2 times, from 57 to 58 days, 0 L, from 59 on.

Calves were fed the liquid diet twice daily at 0700 and 1500 h using a milk bucket. All calves had *ad libitum* access to water and a commercial texturized calf starter (Nuplen Comercializadora, Gómez Palacio, Mexico) consisting of steam-flaked corn, rolled oats, soybean meals, cane molasses, and vitamin and mineral premix. The metabolizable energy (ME) and crude protein (CP) content of the calf starter were 3.08 Mcal/kg and 20%, respectively.

Males from both groups were castrated at 45 days of age using the banding method. The procedure was performed in individual pens by trained personnel, using castration bands (Neogen Corp., Lexington, KY, USA). The castration age reported in this study reflects a common management practice in Mexico and aligns with national data from the U.S. Shivley et al. [22] reported that 27.8% of dairy operations castrate bull calves, with 70% using banding at an average age of 6.3 weeks.

### 2.2. Postweaning Management

**Figure 1:**
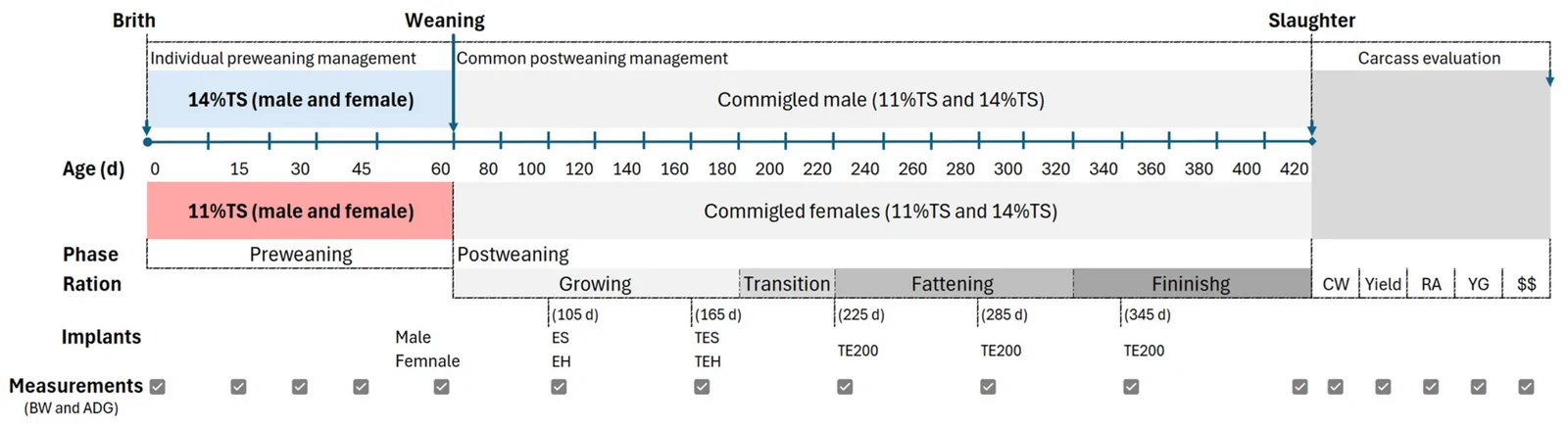
Experimental timeline preweaning nutritional treatments, postweaning management, feeding phases, implant administration, growth measurements, and carcass evaluation of beef-on-dairy calves. Calves received either the 11%TS or 14%TS treatment during the preweaning period (0–60 d). The red and blue shaded bars indicate the 11% TS and 14% TS nutritional treatments, respectively, while arrows indicate the duration of each management phase. Following weaning, calves from both treatments were commingled and managed under identical commercial production conditions until slaughter.

After weaning at 60 days of age, both groups were incorporated into the standard management system of the commercial dairy farm (Figure 1). Therefore, after this age (>60 d), there were no differences in the management between the groups (nutrition, feeding frequency, implants, grouping, and overall handling were the same for all animals). Following weaning, calves from both preweaning treatments (11%TS and 14%TS) were commingled after weaning and grouped according to age and sex as part of the routine management practices of the farm. Animals were housed in soil-surfaced, outdoor 30 × 27 m pens. The concrete feed bunk spanned the width of the pen, with 20 cm of bunk space per animal, and each pen had a center-pen constant-flow concrete water tank. Also, pens had metal shades in the center with 2.2 m^2^ of shade per animal. On arrival at the pens, animals were vaccinated against clostridial diseases (COVEXIN^®^, MSD Salud Animal, Mexico City, Mexico), viral pathogens (BOVILIS^®^, MSD Salud Animal, Mexico City, Mexico).

Pens contained 45 animals as part of the routine commercial management system; therefore, calves were grouped according to both age and sex regardless of preweaning-treatment assignment. The experimental treatments were applied individually during the preweaning period; therefore, the individual calf was considered the experimental unit throughout the study. Calves were separated by age and sex at weaning. The male and female pens were separated by a corridor (6.5 m) to prevent socialization at the joint pen boundaries. These pens were located within the same commercial farm, in a feedlot area designated for beef-on-dairy cattle; therefore, the animals remained in the same facilities from birth until the day of slaughter.

The nutritional management of these animals was carried out in four stages. After weaning and up to six months of age, they consumed a diet consisting of oat hay, flaked corn, and starter concentrate (2.49 Mcal/kg of ME and 16.05% CP and 20.2% NDF). From six to seven and a half months, they received a transition diet consisting of alfalfa hay, flaked corn, TMR-refuse, trace-mineralized salt, confectionery by-products, corn silage, brewer’s spent grain and soybean meal bypass (2.68 Mcal/kg of ME and 10.87% CP and 20.8% NDF), designed to prepare the calves for subsequent diets with a higher grain proportion. From 7.5 to 11 months of age, they were fed a fattening diet consisting of oat hay, flaked corn, trace-mineralized salt, confectionery by-products, corn silage, brewer’s spent grain, urea, cottonseed and calcium salts of fatty acids (2.87 Mcal/kg of ME and 10.57% CP and 21.7% NDF). After 11 months and until they were sold, they consumed a finishing diet consisting of oat hay, flaked corn, trace-mineralized salt, urea, cottonseed and calcium salts of fatty acids (2.98 Mcal/kg of ME and 10.54% CP and 26.9% NDF; Table 1). From weaning to the finishing stage, dry matter intake was offered ad libitum and adjusted weekly per pen based on body weight (BW) and visual assessment (daily bunk readings and refusals feed per pen). After weaning, the TMR of each stage was delivered twice a day, with a proportion of 60 to 70% in the morning and the rest in the afternoon (08:00 to 10:00 and 17:00 to 19:00 h), using a vertical mixer wagon (T3250 Tormex Ind., Torreon. Mexico; 12 tons capacity).

Implants were used in this study to enhance growth and nutrient utilization in calves. All implants used in the study were from the Component line (Elanco Inc., Greenfield, IN, USA), and the implant protocol was as follows: Implants were administered at 105, and 165 days of age, differing between male and female groups. At 105 days of age, males from both treatment groups received a dose of Component ES implant (Progesterone 200 mg, Estradiol Benzoate 20 mg, Tylosin Tartrate 29 mg). At the same age (105 days), females from both treatment groups received a dose of Component EH implant (Testosterone Propionate 200 mg, Estradiol Benzoate 20 mg, Tylosin Tartrate 29 mg). At 165 days of age, males from both treatment groups received a dose of Component TES implant (Trenbolone Acetate 120 mg, Estradiol Benzoate 24 mg, Tylosin Tartrate 29 mg), while females from both treatment groups at the same age (165 days) received a dose of Component TEH implant (Trenbolone Acetate 140 mg, Estradiol Benzoate 14 mg, Tylosin Tartrate 29 mg). Following these implants, all animals (males and females) received three doses of Component TE200 implants (Trenbolone Acetate 200 mg, Estradiol Benzoate 20 mg, Tylosin Tartrate 29 mg) at 60-day intervals, administered at 225, 285, and 345 days of age.

The implants were administered by trained personnel in accordance with the product guidelines (Component Elanco Inc., Greenfield, IN, USA), which include proper placement and sanitization of the application area. The weighing and implantation procedures during the postweaning stage were carried out using a livestock squeeze chute (1500 scale-chute by basculas NuevoLeón, Monterrey, Mexico, with a capacity of 1.5-tons and an accuracy of 0.2 g).

Animal selection for slaughter was carried out by the commercial dairy farm based on weight, age, average daily gain, and visual assessment, as well as the sale lot to which the animals were assigned (four types of lots were used, categorized by animal quality and weight). Data were collected at the time of slaughter at the commercial packing plant, including live weight, hot carcass weight, and yield. The plant is certified under the Federal Inspection Type (TIF) program by Mexico’s National Agro-Alimentary Health, Safety, and Quality Service (SENASICA). Carcass quality data were digitally collected using the VGB 2000 system (Marel LLC., Des Moines, IA, USA), which is included among the USDA Approved Instruments for Augmenting Beef Grading [23].

A total of 80 calves were enrolled in the study (40 calves per treatment), and all animals remained in the experiment through slaughter. Consequently, all enrolled calves were included in the growth performance, carcass, and economic analyses. The Holstein × beef calves originated from the farm’s commercial beef-on-dairy breeding program using commercial beef sire genetics.

### 2.3. Statistical Analysis

Before analysis, all data were checked for normality using the UNIVARIATE procedure in SAS (SAS 9.4, SAS Institute Inc., Cary, NC, USA). Repeated measurements of body weight (BW) and cumulative average daily gain (cumulative ADG) were analyzed using generalized linear mixed models (GLMM) implemented in the GLIMMIX procedure of SAS. The model included treatment (11% TS and 14% TS), sex, age, and their two- and three-way interactions (treatment × sex, treatment × age, sex × age, and treatment × sex × age) as fixed effects. Calf identification was included as a random effect to account for repeated observations within animals throughout the study period:

Y_ijkl_ = μ + T_i_ + S_j_ + A_k_ + (T × S)_ij_ + (T × A)_ik_ + (S × A)_jk_ + (T × S×A)_ijk_ + ID_l_ + _εijkl_

where

- Y_ijkl_ = body weight (BW) or average daily gain (ADG);
- μ = overall mean;
- T_i_ = fixed effect of treatment (11% TS or 14% TS);
- S_j_ = fixed effect of sex;
- A_k_ = fixed effect of age;
- (T × S)_ij_ = treatment × sex interaction;
- (T × A)_ik_ = treatment × age interaction;
- (S × A)_jk_ = sex × age interaction;
- (T × S×A)_ijk_ = treatment × sex × age interaction;
- ID_l_ = random effect of calf identification;
- ε_ijkl_ = residual error.

For categorical variables (lot type and beef grading), in addition to the MIXED procedure, the FREQ procedure in SAS version 9.4 was used to generate contingency tables and assess group distributions. In the specific case of the beef grading × group × sex interaction, the GLIMMIX procedure in SAS version 9.4 was used with a binomial logit model, with multiple comparisons via Tukey’s HSD test, targeting the highest beef grading category (Choice).

For variables such as age at sale and age adjusted to reach 500 kg BW, the LIFETEST procedure in SAS version 9.4 was used with the Log-Rank test. Age at sale represented the observed age at which each animal was harvested (slaughter). In contrast, the estimated age to reach 500 kg BW was calculated individually as follows:

$$\mathrm{Estimated}\;\mathrm{age}\;\mathrm{at}\;500\;\mathrm{kg}\;\mathrm{BW}\;=\;\frac{\left(500\;kg-BW\;birth\right)}{Total\;ADG}$$

where total ADG represents the average daily gain over the entire production period. This variable represents the estimated age required to attain 500 kg BW based on the individual total ADG. The 500 kg threshold was selected because it approximated the average slaughter weight of calves in the 11% TS treatment, providing a common weight-based endpoint for treatment comparisons.

To determine the influence of pre-weaning nutritional planes on final BW and income per animal, bivariate linear regression analyses were conducted using the REG procedure in SAS version 9.4. Specifically, correlations were evaluated between 60-day cumulative ADG × final weight × group, as well as between 60-day cumulative ADG × economic value per animal. A similar analysis was conducted to assess the effect of cumulative ADG on lot type, using generalized linear regression via the REG procedure in SAS version 9.4.

Carcass market value was determined using two approaches:

First, based on the actual sale price in the Mexican market, calculated as:

Individual price (USD/animal) = USD price per kg of carcass × carcass weight (kg)

Second, considering the estimated market value in the U.S. beef market, based on USDA beef grading, which is being adopted by the Mexican premium beef market but was not yet implemented at the time of sale. The estimated U.S. market price was calculated using the USDA beef carcass price equivalent index value (600–900 pounds) as of 14 September 2025, [24]. All carcasses in the study fell within this USDA reference range 600–900 pounds (272–408 kg). For Choice and Select grades, the report provides prices in dollars per hundredweight (CWT): Individual U.S. market price ($/animal) = USD price per pound = (CWT of the corresponding grade/100) × carcass weight (pounds).

Total revenue per group was calculated under both scenarios using the following breakdowns: sum per group, then further differentiated by sex, and finally by beef grading. Additional feeding costs associated with enhanced nutritional treatment were not included in these calculations. Finally, to evaluate the overall integrated production response to preweaning nutritional plane, a multivariate analysis of variance (MANOVA) was conducted including slaughter weight, carcass weight, dressing percentage, ribeye area, yield grade, lot classification, and beef grading as response variables. Treatment, sex, and treatment × sex interaction were included as fixed effects.

## 3. Results

The results of this research are presented in two sections. The first section discusses the short-term effects of nutrient intakes from two liquid diets with 14% or 11% TS during the preweaning phase, and how these affected calf development and performance parameters. In the second section, development and performance parameters of the calves were measured after weaning, throughout the growth, fattening, and finishing stages of the cattle. Additionally, carcass quality parameters were assessed to determine whether the nutrient intakes provided during the preweaning phase had any long-term effects.

### 3.1. Preweaning Phase Results

**Figure 2:**
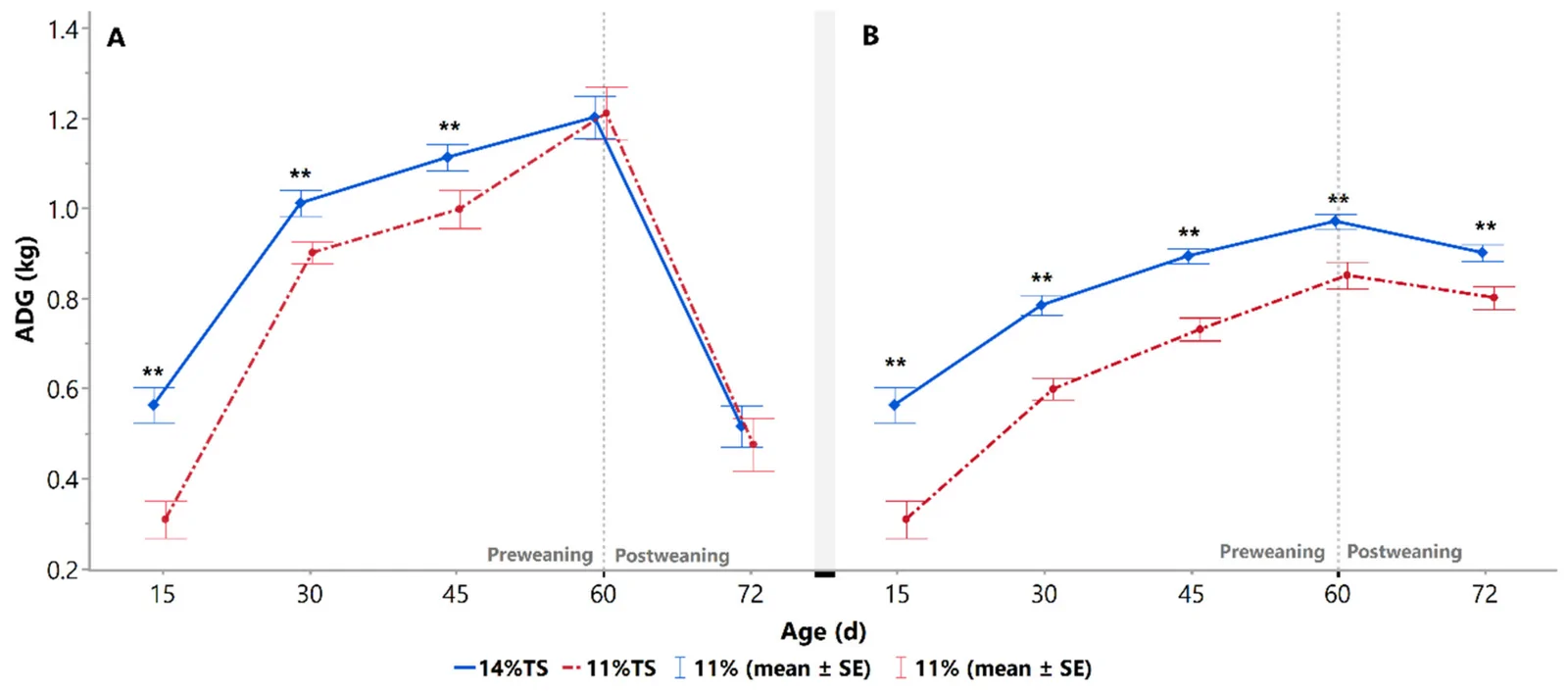
Average daily gain (ADG) of Holstein × beef calves fed milk containing 11% or 14% total solids. (**A**) ADG evaluated every 15 days from 0 to 72 days of age. (**B**) Cumulative ADG from 15 to 72 days of age. Data are presented as means ± standard error (SE). ** *p* < 0.01. Weaning was concluded at 60 days, after which calves were transferred to group housing.

The enhanced feeding plan (14%TS) design (450 L at 14%TS = 63 kg DM) provided the calves with an average additional intake of +225 g TS per day (63–49.5/60), compared to the control group (450 L at 11%TS = 49.5 kg DM). Increasing total solids in the milk offered to preweaning beef-on-dairy calves improves early growth performance (Table 2; Figure 2). The greatest difference between groups was observed during the first evaluation at 15 days of age, where the 14%TS group showed (>0.200 kg/d) a significantly greater ADG (*p* < 0.001) compared to the 11%TS group (0.563 ± 0.04 vs. 0.302 ± 0.04 kg/d). This effect is attributed to the greater intake of milk solids, as starter intake at that age is minimal; calves fed the enhanced diet (14%TS) exhibited better early development. Similarly, at 30 and 45 days of age, the enhanced diet group (14%TS) continued to show greater growth (*p* < 0.004 and *p* < 0.002, respectively), with ADG values (1.011 ± 0.03 vs. 0.901 ± 0.03 kg/d and 1.113 ± 0.03 vs. 0.998 ± 0.04 kg/d, respectively) compared to the conventional milk group. At weaning, the 14%TS group presented a greater cumulative ADG over 60 days (0.971 ± 0.02 vs. 0.851 ± 0.03 kg/d; *p* < 0.01). This resulted in a greater weaning body weight for the 14%TS group (98.01 ± 1.29 vs. 93.28 ± 1.85 kg; *p* < 0.05) compared with the 11%TS group.

By the end of the pre-weaning phase, no significant differences in ADG were observed between sexes within the control group (*p* = 0.91), with males and females performing similarly (0.847 ± 0.03 vs. 0.854 ± 0.05 g/d, respectively). Likewise, in the enhanced diet group (14%TS), no sex-based differences were observed (*p* = 0.16), although a trend toward greater ADG was noted in females (0.993 ± 0.02 vs. 0.948 ± 0.02 g/d). Overall, during the preweaning phase, beef-on-dairy calves show no sex-related differences in development.

### 3.2. Postweaning Phase Results

The first postweaning evaluation was conducted at 72 days of age to evaluate the transition from milk and starter-based feeding to a forage and concentrate-based ration. After weaning, animals were no longer segregated by nutritional group (14%TS or 11%TS), only by sex, and both groups were housed together; as a result, it was not possible to determine differences in dry matter intake by group. However, the evaluation at 72 days showed that the 14%TS group had better performance (*p* < 0.002), with greater cumulative ADG (0.901 ± 0.02 vs. 0.801 ± 0.03 kg/d) and a more consistent ADG distribution at that age (Figure 1). Some animals in the control group even exhibited low or negative ADG values. BW at 72 days of age was also greater by 4.47 kg in the 14%TS group (104.59 ± 10.70 vs. 100.12 ± 12.961 kg) compared to the 11%TS group.

**Figure 3:**
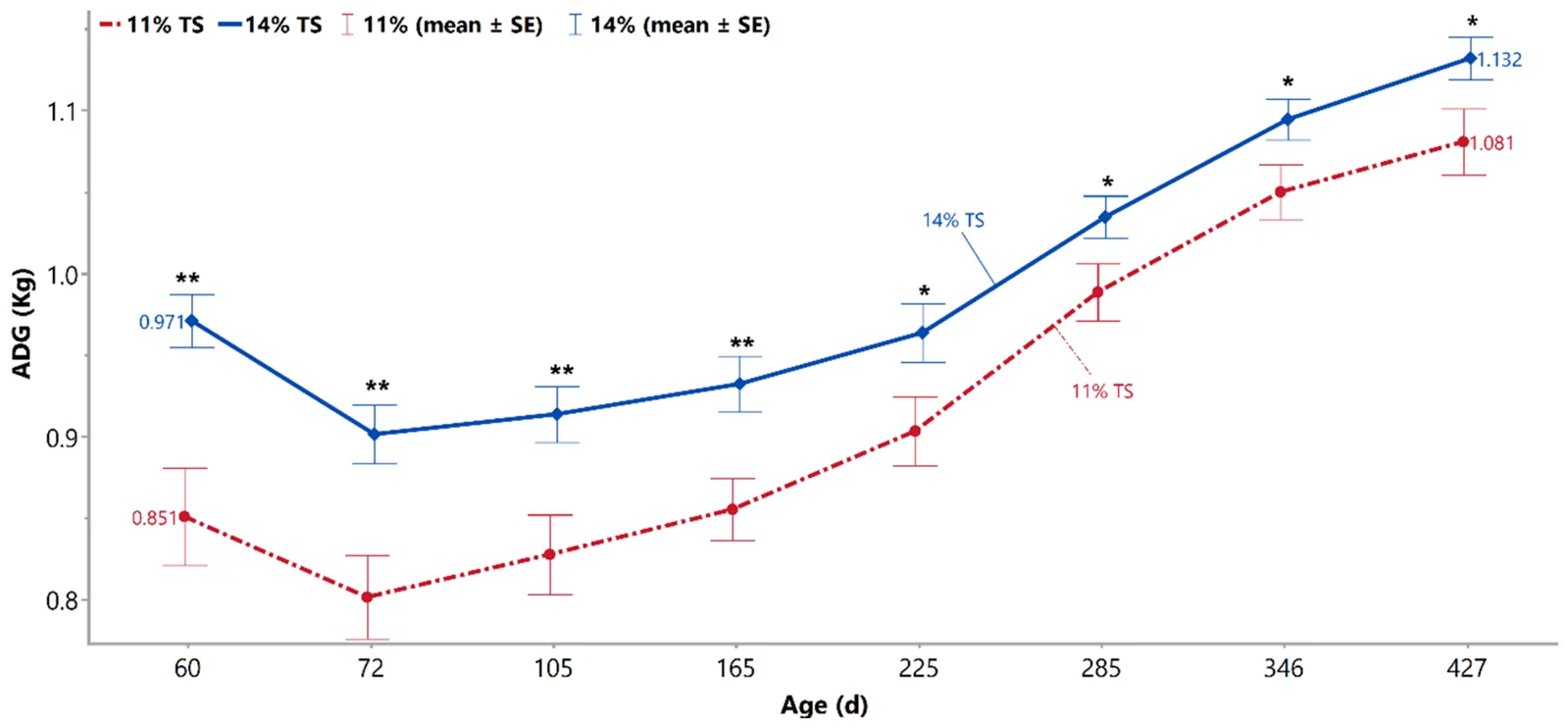
Postweaning cumulative average daily gain (ADG) of Holstein × beef calves fed milk containing 11% or 14% total solids during the preweaning period. Cumulative ADG from 60 to 427 days of age. Data are presented as means ± standard error (SE). * *p* < 0.05; ** *p* < 0.01. Weaning was concluded at 60 days, after which growth performance was monitored until slaughter.

The most effective way to evaluate the group’s response to the nutrient supply received in the previous phase is by analyzing the cumulative ADG up to slaughter. (Figure 2). When examining postweaning ADG, the gap between the two groups gradually narrowed over time. After weaning, the difference was most pronounced (*p* < 0.01) and persisted until 165 days of age. After that point, the difference remains (*p* < 0.05), and the final evaluation still favored the 14%TS group over the 11%TS group (Table 2; Figure 3). The first implant after weaning was administered at 105 days of age. At that point, cumulative ADG was greater (*p* < 0.01) in the 14%TS group (0.914 ± 0.02 vs. 0.827 ± 0.02 kg/d) compared to the control group. At 165 days, the 14%TS group continued to show superior cumulative ADG (*p* < 0.01; 0.932 ± 0.02 vs. 0.855 ± 0.02 kg/d). In the following three evaluations, cumulative ADG remained greater in the 14%TS group: +60 g at 225 days (*p* < 0.05), +46 g at 285 days (*p* < 0.05) and +45 g at 346 days (*p* < 0.05). For the final ADG assessment at slaughter, the 14%TS group outperformed (*p* < 0.05) the control group (1.132 ± 0.01 vs. 1.081 ± 0.02 kg/d). This accumulated ADG advantage was associated with greater final BW at slaughter, with the 14%TS group being superior (*p* < 0.05) to the 11%TS group (520.37 ± 3.60 vs. 502.92 ± 7.36 kg). Unlike the preweaning stage, sex differences were observed in cumulative ADG at sale, with males (*p* < 0.01) outperforming females (1.161 ± 0.02 vs. 1.051 ± 0.02 kg/d). Within the groups (14%TS and 11%TS), males also showed better performance (*p* < 0.05 and *p* < 0.01, respectively) compared to females of the same group (14%TS: 1.169 ± 0.02 vs. 1.094 ± 0.02 kg/d and 11%TS: 1.153 ± 0.02 vs. 1.008 ± 0.02 kg/d). Males in the 14%TS group had the highest cumulative ADG. When evaluating the correlation between cumulative ADG at 60 days and final weight, a strong correlation was observed in the 14%TS group (*p* < 0.01), while no such correlation was observed in the 11%TS group (*p* = 0.748). This response indicated that, in the enhanced feeding group (14%TS), every 100 g increase in preweaning cumulative ADG is associated with a 9.75 kg increase in final body weight.

**Figure 4:**
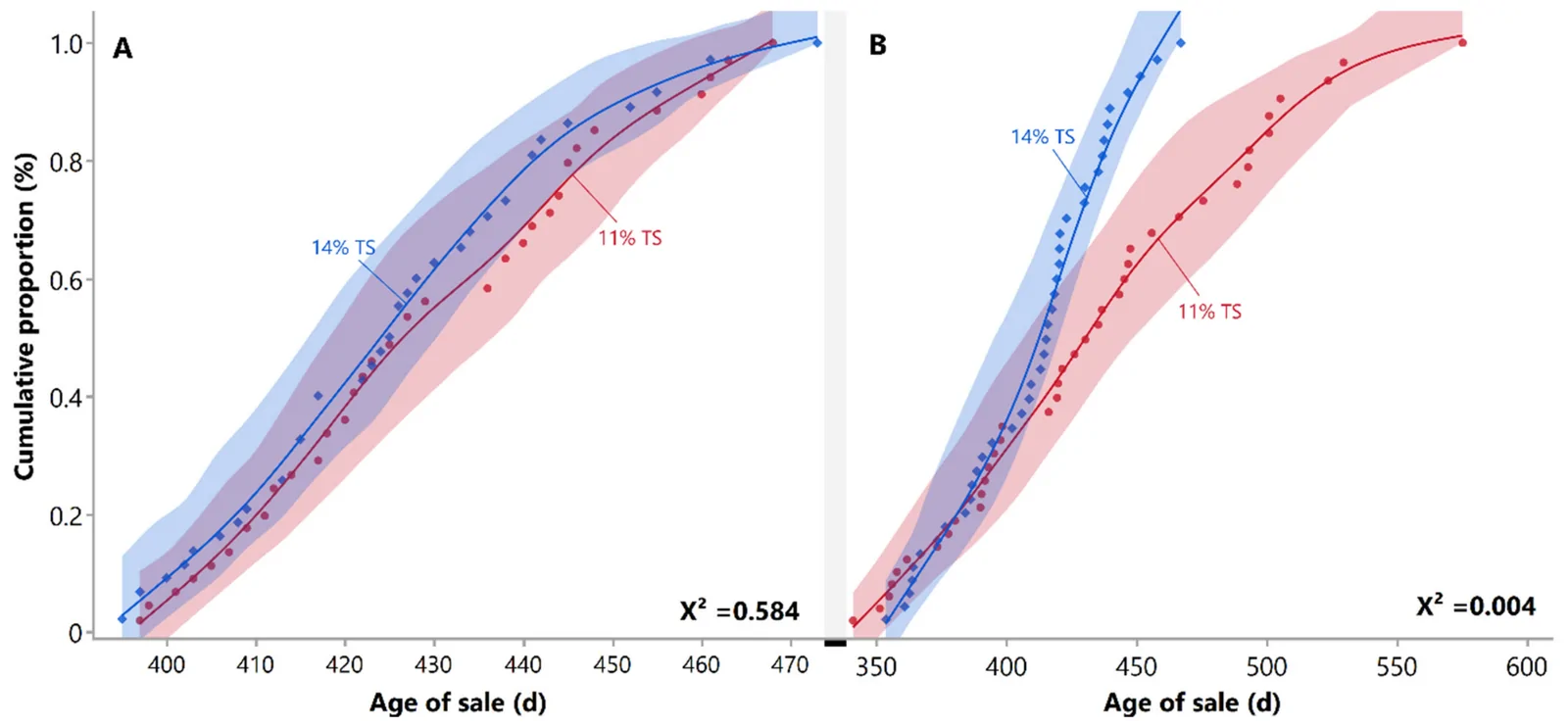
Age of sale by group of Holstein × beef heifers and steers. Kaplan–Meier survival curves with confidence intervals estimated via isolated spline adjustment, applied to age at sale (**A**) and age adjusted (**B**) to reach 500 kg (500 kg − birth weight/individual total ADG). The threshold of 500 kg was selected because the average sale weight of the control group was close to this value, representing the theoretical age at sale under a uniform weight-based criteria. The blue and red curves represent the 14% TS and 11% TS treatments, respectively. Shaded areas indicate 95% confidence intervals, and dots represent individual observations.

No significant differences were observed in age at sale. However, calves in the 14%TS group were sold 2.36 days earlier on average (425.5 ± 3.0 vs. 427.9 ± 3.0 days). Since final weight did differ, the age of the 14%TS group was adjusted to determine when they reached the average sale weight of the 11%TS group. This analysis showed that the 14%TS group reached the control group’s average sale weight (408.6 ± 29.2 vs. 430.1 ± 56.7 days) at a younger age (*p* < 0.05; Figure 4).

**Figure 5:**
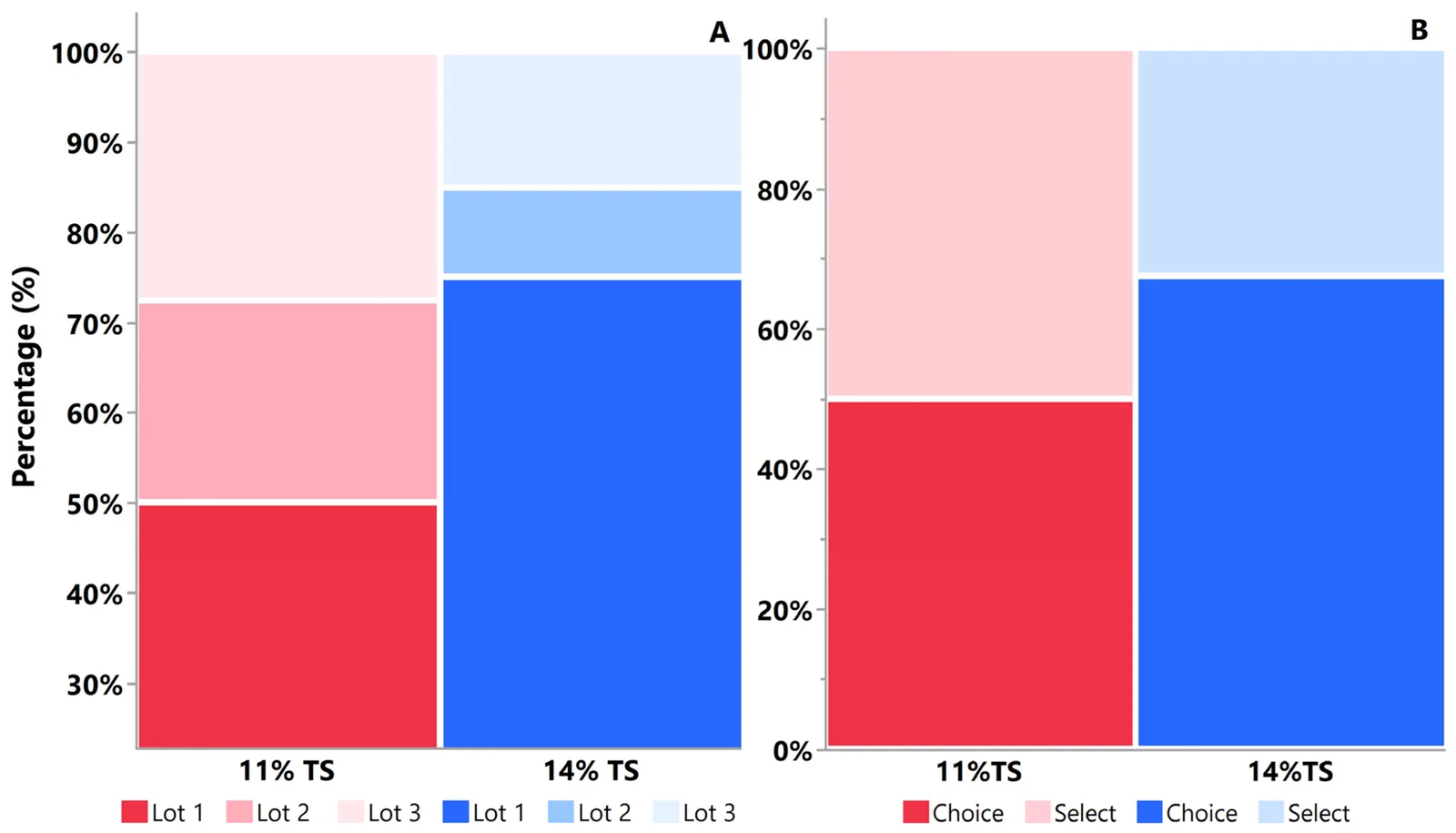
Distribution of lot type (**A**) and USDA beef grading (**B**) in Holstein × beef heifers and steers fed diets containing 11% or 14% TS during the preweaning period. Panel (**A**) shows the proportion of animals classified into Lot 1, Lot 2, and Lot 3 within each group. Panel (**B**) shows the proportion of carcasses graded as Choice or Select.

Lot assignment and animal selection for slaughter were carried out by the commercial farm, based on development criteria (BW, cumulative ADG and age); this process was not random and was not part of the experimental design. A strong correlation was observed (*p* < 0.01) between lot type and cumulative ADG. Animals with greater cumulative ADG were assigned to higher-quality lots. Group analysis showed that the enhanced feeding group had a higher (*p* < 0.05) proportion of animals in premium lots (Lot 1: 75% vs. 50%, Lot 2: 10% vs. 23% and Lot 3: 15% vs. 28%; Table 3; Figure 5). When evaluating lot type by sex, a difference is observed (*p* < 0.01), with a greater proportion of males found in higher-quality lots (1.175 ± 0.07 vs. 2.00 ± 0.14-point male and female), males also showed better performance compared to females of the same group (14%TS: 1.10 ± 0.07 vs. 1.70 ± 0.20 point and 11%TS: 1.25 ± 0.12 vs. 2.30 ± 0.18 point). Males in the 14%TS group had the highest lot type classification. The average value per lot type under actual Mexican market conditions was calculated by summing the purchase value of animals ($animal = $/kg carcass × kg carcass), grouped by lot type: for Lot 3 (lower quality): USD1335.94, Lot 2: USD1435.21 (+USD 99.37) and Lot 1: USD 1615.76 (+USD 180.45 vs. Lot 2). Due to the greater number of animals (*p* < 0.05) from the 14%TS group in premium lots, this group showed a greater economic advantage at sale (*p* < 0.01), with a total benefit of over USD 3300 compared to the 11%TS group (USD 1569.45 ± 16.11 vs. USD 14864.58 ± 30.20 per animal).

**Figure 6:**
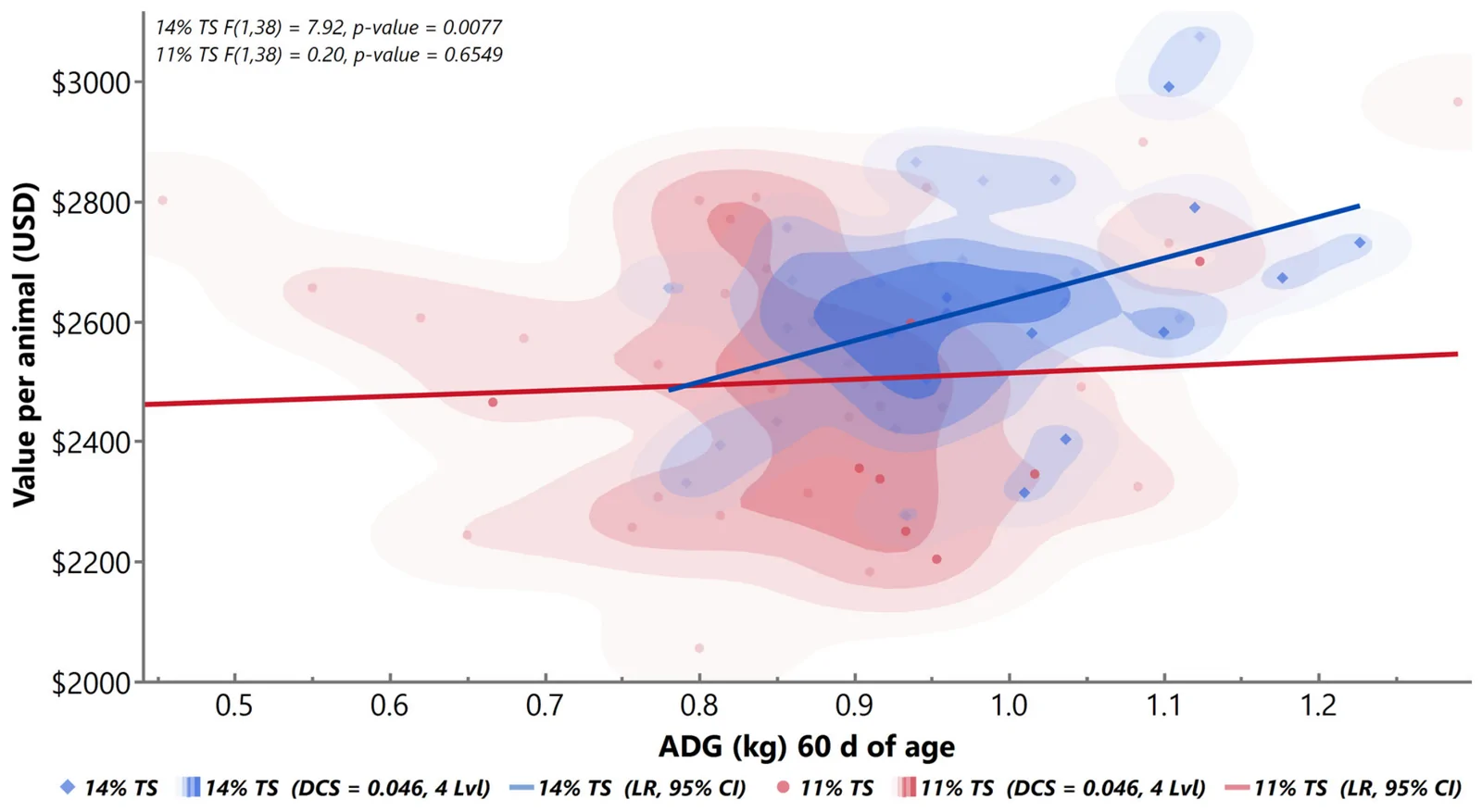
Relationship between average daily gain (cumulative ADG, kg at 60 d of age) and economic value per animal (USD) in Holstein × beef heifers and steers fed diets containing 11% or 14% TS during the preweaning period. Scatter plots with density contours (DCS = 0.046, 4 levels) and linear regression lines (LR, 95% CI) are shown for each group.

Regarding carcass grading, data from the VGB2000 system showed no significant differences between groups, though the 14%TS group tended toward a greater number of animals classified as “Choice” (67.5% and 32.5% or 27 and 13 animals), while the 11%TS group had equal “Select and Choice” classifications (50% and 50% or 20 and 20 animals; Table 4; Figure 5). No significant differences were observed in carcass weight, yield, or yield grade between groups. The ribeye area showed a trend toward being larger in the 14%TS group compared to the 11%TS group (*p* = 0.068). Although these parameters were not statistically significant, favorable numerical differences were observed in the 14%TS group, including greater yield (61.25% vs. 60.12%), total carcass weight (12,791.2 vs. 12,480.4 kg), larger ribeye area (79.83 vs. 78.43 cm^2^) and a lower yield grade indicator (2.79 vs. 2.96 YG). These numerical responses are associated with economic advantage for the 14%TS group (Figure 6). USDA Yield Grades (YG) estimate beef carcass cutability, which is defined as the combined yield of closely trimmed, boneless retail cuts (%CTBRC) from the round, loin, rib and chuck.; the lower the YG value, the higher %CTBRC and total retail cuts (% Total Yield carcasses). To evaluate the overall integrated production response associated with preweaning nutritional plane, a multivariate analysis of variance (MANOVA) was conducted including slaughter weight, carcass weight, dressing percentage, ribeye area, yield grade, lot classification, and beef grading as response variables. Although individual carcass traits showed only numerical differences between treatments, the multivariate analysis detected a significant overall treatment effect (*p* < 0.05), indicating differences in the combined production outcomes of calves receiving 14%TS and 11%TS during the preweaning period. Under the USDA beef-grading market scenario, the evaluation of average per animal (USD) by group× sex × beef grading showed no significant difference (*p* > 0.1) between males and females in the 14%TS group, indicating similar revenue (USD 2695.32 ± 41.5 vs. USD 2661.43 ± 40.8, male and female). Similarly, the average per animal (USD) by group× sex, showed no difference (*p* > 0.1) between males and females in the 14%TS group (USD 2630.71 ± 25.1 vs. USD 2602.34 ± 39.9). However, total income by sex was higher for males (USD 103,791.80 vs. USD 100,839.61) than for females, with an average income per animal of USD 73.80. This difference was evident regardless of the number of animals classified as Choice or Select (20 and 20 vs. 27 and 13, respectively). The 11%TS female group showed the lowest performance of all calves. The groups with enhanced nutrition performed better (*p* < 0.05) than those who were fed a conventional diet (USD 2616.53 ± 26.9 vs. USD 2499.26 ± 39.4), resulting in a total benefit of over USD 4600. In the group fed 14% TS, a positive association was observed between cumulative ADG at weaning (60 d) and the economic value per animal (*p* < 0.01), whereas in the group fed 11% TS no such association was observed (*p* = 0.65; Figure 6).

## 4. Discussion

### 4.1. Preweaning Phase Discussion

Calves fed 14%TS diet consumed an average 225 g more TS per day, compared to the control group (11%TS). This was reflected in the calves’ ADG and resulted in greater weaning weight. The most significant difference in ADG between groups was observed at the first evaluation (15 days), when the 14%TS group showed an 86% greater ADG (+263 g/day) than the control group. These findings highlight the importance of enhanced nutrition in supplying essential nutrients during the early days of life. Previous studies confirm that enhanced feeding programs increase nutrient intake from liquid diets (protein, lactose, fat, and total solids), improve preweaning health [17], do not interfere with rumen development [25], enhance feed efficiency [26], and strengthen immune responses postweaning [27]. In the present study, calves in the control group showed lower ADG at 15 days, with greater dispersion, and some even exhibited negative ADG.

The cumulative ADG at weaning was greater in the enhanced feeding group (971 g/day, or +120 g/day), resulting in a greater weaning weight (+7.20 kg) than in the control group. These outcomes are explained by the strong positive relationship between enhanced nutrition (higher CP and ME) and preweaning ADG [28]. These findings are consistent with previous studies showing that calves receiving enhanced nutrition had improved ADG and weaning weight [9,26,29].

In the United States, there is greater diversification in preweaning calf management and nutrition systems [30]. However, in Mexico, it remains common practice to feed calves during the preweaning phase using the so-called conventional system, which is defined as offering or restricting milk intake to 10% of BW [31,32], typically using liquid diets containing 12% total solids. This approach has been used for decades not only in Mexico but also worldwide [9,10,13,17,30]. This feeding scheme allows weaning at approximately 60 days of age and reduces the overall cost of calf nutrition [10,33]. However, these conventional programs have been shown to reduce weight gain in calves [34,35] and negatively impact calf welfare [36,37]. Therefore, providing enhanced nutrition plans during the preweaning phase increases ADG and weaning weight, as confirmed by the results of the present study.

Various strategies can be used to enhance nutrition, either by increasing the concentration of total solids in the same volume of milk [38,39], or by increasing the volume of milk or milk replacer with moderately elevated solids content [32]. Still, the results consistently show improvements in growth performance.

In the study by Azevedo et al. [38], the 20.4% TS group received a total dry matter intake during the preweaning phase comparable to that of the 14%TS group in the present study, and their ADG at approximately 60 days of age was similar (903 vs. 971 g/day). Likewise, in the study by Chapelain et al. [31], the MR-H group (milk replacer at 16.2% TS) had a cumulative ADG at week 10 comparable to the 14%TS group at 72 days in the present study (998 vs. 901 g/day), considering that the MR-H group underwent a more gradual and extended weaning process. These findings illustrate four distinct approaches for delivering enhanced nutrition to preweaning-age calves. Regardless of the strategy used, calves receiving enhanced nutrition consistently demonstrated improved growth performance compared to their respective control groups.

### 4.2. Postweaning Phase Discussion

The postweaning (long-term) effects of enhanced preweaning nutrition programs remain a controversial topic, particularly regarding the extent of their impact and the best way to quantify them. Moreover, many studies fail to include postweaning data. Kertz [18] recommends reporting outcomes at least 2 to 4 weeks after weaning to assess any residual effects of the preweaning program. There is evidence from dairy heifers that supports the long-term benefits of enhanced preweaning nutrition programs on reproductive performance [13,14], milk yield [14,15,16], and productive lifespan [14,17] as well as on postweaning effects. Rosenberger et al. [40] observed that calves fed greater amounts of milk maintained an advantage in weight gain during and after weaning when encouraged to transition to solid feed. Similarly, other authors have reported that calves fed increasing levels of milk replacer or enhanced replacer during the preweaning period grew faster after weaning [41,42].

Few studies have reported the long-term effects of enhanced preweaning nutrition programs in Holstein × beef calves. For example, Azevedo et al. [37,38] documented growth performance in heifer calves at 90 and 55 days of age. Information becomes even scarcer when considering intensive commercial dairy systems [10]. It has observed that the long-term effects of high total solids concentration in milk or milk replacer on growth performance in dairy calves tend to diminish after 180 days of age. Similarly, Leal et al. [42] reported that by 330 days of age, BW differences were no longer significant between calves fed higher milk volumes during the preweaning period and those receiving conventional volumes (4 L/day). These findings contrast with the results of the present study, in which the cumulative ADG difference between groups gradually decreased over time (−5.6 g/month). Still, this reduction was not enough to eliminate the performance advantage of the enhanced nutrition group, which maintained significantly greater ADG (*p* < 0.05) and BW (*p* < 0.05) at the final evaluation at 427 days (+17.5 kg for the 14%TS group). This response suggests that the growth advantage established during the preweaning phase persisted throughout the production period.

Another way to reaffirm the influence of preweaning nutrition level is through the correlation between weaning cumulative ADG and final BW, which was strongly significant (*p* < 0.01) in the enhanced nutrition group, but not in the control group. These results differ from those reported by Silva et al. [43], who found that calves with lower weaning weights exhibited compensatory growth, resulting in similar final weights to those of calves receiving enhanced nutrition. In one study on Holstein × beef cattle in an organic system, Vestergaard et al. [44] reported ADG and final weight at 17 months of age (1.039 kg/day and 575 kg). In another system, a U.S. feedlot study [45] reported ADG and final weight in beef × dairy cattle (1.51 kg/day and 617 kg) although the reported ADG only reflected the feedlot period, with no data on cumulative ADG or animal age. In comparison, beef × cross-dairy cattle raised in 100% pasture systems had an ADG of 665 g/day and a final weight of 662 kg at 784 days [46].

Although carcass traits did not show statistically significant differences between groups, a positive trend was observed in the enhanced nutrition group, which contributed to a greater proportion of animals classified into higher-quality lots (+25% in lot type 1) and greater numerical proportion of carcasses classified as Choice (67.5%), as determined by USDA-approved automatic grading. From January to September 2025, the national average of carcasses graded as Choice in the United States was 72.75% [47]. The 14%TS group approached this benchmark, considering that the study was conducted in the Mexican market, where target slaughter weights are lower and the premium beef segment is still developing. Unlike the U.S., Mexico’s premium beef market does not yet differentiate prices based on carcass grading. In contrast, the U.S. market shows a marked price difference between “Choice” and “Select” carcasses (272–408 kg), with an estimated USD 170 advantage per “Choice” carcass [24]. This highlights how market structure influences the economic interpretation of carcass quality outcomes. Furthermore, in beef × dairy cattle with higher target weights, the proportion of carcasses graded as Choice is typically higher, as reported by Foraker et al. [48], with 81.1% graded as Choice or higher.

Vestergaard et al. [44] and Coleman et al. [45] reported carcass grading scales that differ from USDA standards, making direct comparisons difficult. Regarding ribeye area, Coleman et al. [45] reported 81.93 cm^2^, while Foraker et al. [48] reported 92.2 cm^2^, compared to 80.18 cm^2^ observed in the 14%TS group of the present study. Regarding sex differences, this study found that male calves exhibited superior growth parameters (ADG, BW, and carcass weight), whereas female calves achieved better carcass grading outcomes. These findings are consistent with previous reports [48,49]. Although females tend to achieve higher USDA beef-grading scores, the observed differences in ADG, BW, carcass weight and yield resulted in a comparative economic advantage averaging USD 73 per animal compared to females. Under the USDA beef-grading market scenario, the economic analysis between the nutritional groups revealed a revenue advantage (USD 117 per animal) for the enhanced nutrition group (*p* < 0.01) compared to the control group, representing a total difference of over USD 4600.

Overall, Holstein × beef male and female calves included in this study showed good rearing performance under the management conditions employed, comparable to Holstein steers managed on the same farm where the study was conducted. Under the conditions of this study, no additional care or management adjustments were required for Holstein × beef cattle. Additionally, Holstein × beef heifer calves appeared to show distinct consumption patterns, with greater appetite and an apparent higher intake in milk volume and total solids. Although this observation was not systematically evaluated, it was consistently observed throughout the study and suggests a promising line of inquiry for future research.

## 5. Conclusions

The results of this study demonstrate that feeding Holstein × beef calves under an enhanced nutrition plan (14% TS) improves growth performance (ADG and BW) in the short term (at weaning) and sustained long-term advantages in cumulative ADG and final BW. These improvements were reflected in favorable numerical responses in carcass traits and USDA beef grading and, in turn, translated into a revenue advantage per animal compared to the control group. Overall, these findings contribute to improving the profitability of the dairy industry and support the strategic implementation of the beef-on-dairy model. Furthermore, they highlight the advantages of adopting a payment system based on USDA beef grading, which recognizes the commercial value of carcasses with superior characteristics.

## Figures and Tables

**Table 1:** Nutritional characteristics of the preweaning starter and postweaning feeding phases used in the study.

Parameter/Phase	Starter ^5^	Growing	Transition	Fattening	Finishing
Age (d)	0–60	60–180	180–225	225–330	>330
ME (Mcal/kg) ^1^	3.08	2.49	2.68	2.87	2.98
CP (%) ^2^	20.00	16.05	10.87	10.57	10.54
NDF ^3^	15.8	20.2	20.8	21.7	26.9

^1^ ME = metabolizable energy. ^2^ CP = crude protein. ^3^ NDF = neutral detergent fiber. ^4^ Values are expressed on a DM basis. ^5^ Preweaning period (0–60 d), calves fed milk containing 11%TS or 14%TS liquid diet; starter intake was offered ad libitum. ^6^ Postweaning period, all calves were managed under identical nutritional and management conditions.

**Table 2:** Growth performance of Holstein × beef calves during preweaning and postweaning phases when fed milk containing 11% or 14% total solids in the preweaning phase (<60 days).

	**Preweaning ADG (kg)**				
Age (day) ^1^	14%TS		11%TS		*p*-value
15	0.563 ± 0.038 ^a^		0.302 ± 0.041 ^b^		0.0001
30	1.011 ± 0.028 ^a^		0.901 ± 0.024 ^b^		0.0043
45	1.113 ± 0.029 ^a^		0.998 ± 0.042 ^b^		0.0029
60	0.971 ± 0.016 ^a^		0.851 ± 0.029 ^b^		0.0007
Weight	98.0 ± 1.29 ^a^		93.2 ± 1.85 ^b^		0.040
By sex ^3^	Male	Female	Male	Female	
ADG (kg)	0.948 ± 0.023 ^a^	0.993 ± 0.021 ^a^	0.847 ± 0.033 ^b^	0.854 ± 0.049 ^b^	
Weight	96.9 ± 1.92 ^a^	99.1 ± 1.75 ^a^	95.8 ± 10.32 ^b^	90.7 ± 2.86 ^b^	
Age (day) ^2^	**Postweaning cumulative ADG (kg)**				
72	0.901 ± 0.017 ^a^		0.801 ± 0.025 ^b^		0.002
105	0.913 ± 0.018 ^a^		0.827 ± 0.024 ^b^		0.005
165	0.932 ± 0.017 ^a^		0.855 ± 0.019 ^b^		0.004
225	0.963 ± 0.017 ^a^		0.903 ± 0.021 ^b^		0.033
285	1.034 ± 0.012 ^a^		0.988 ± 0.017 ^b^		0.037
346	1.094 ± 0.012 ^a^		1.050 ± 0.016 ^b^		0.036
427	1.132 ± 0.013 ^a^		1.080 ± 0.020 ^b^		0.038
Weight	520.4 ± 3.60 ^a^		502.9 ± 7.36 ^b^		0.036
By sex ^3^	Male	Female	Male	Female	
ADG (kg)	1.169 ± 0.016 ^a^	1.094 ± 0.016 ^b^	1.153 ± 0.025 ^a^	1.008 ± 0.022 ^c^	
Weight	525.5 ± 4.64 ^a^	515.2 ± 5.39 ^a^	521.8 ± 10.13 ^a^	484.0 ± 9.07 ^b^	

^1^ For the preweaning phase, ADG was assessed at the following intervals: 0 to 15 days (15 d), 15 to 30 days (30 d), 30 to 45 days (45 d), and 60 days (cumulative ADG from 0 to 60 days). ^2^ For the postweaning phase, only cumulative ADG from day 0 to each evaluation point was used to assess the effect of different nutritional levels over time in the preweaning phase. ^3^ Least squares mean by sex and weight that do not share a common superscript letter (a, b) differ significantly (*p* < 0.05) according to Tukey’s HSD test.

**Table 3:** Lot type, carcass traits and economic analysis of Holstein × beef (heifers and steers) fed milk containing 11% or 14% total solids in the preweaning phase, based on actual Mexican market sale prices.

**Lot Type Traits**							
ITEM	14% TS			11% TS			*p*-value
Lot type	1.400 ± 0.117 ^a^			1.775 ± 0.861 ^b^			0.040
Lot type (n) ^1^	Lt-1	Lt-2	Lt-3	Lt-1	Lt-2	Lt-3	
	30	4	6	20	9	11	
By sex	Male	Female		Male		Female	
Lot type (point) ^2^	1.10 ± 0.07 ^a^	1.70 ± 0.20 ^b^		1.25 ± 0.55 ^bc^		2.30 ± 0.80 ^c^	
Lot type USD ^3^	Lot 1			Lot 2		Lot 3	
	$1615.76			$1435.31		$1335.94	
**Carcass traits**							
ITEM	14%TS ^4^			11%TS ^4^			*p*-value
Hot carcass weight (kg)	319.7 ± 3.07 ^a^			312.0 ± 6.28 ^a^			0.2498
Dressing percentage (%)	61.25 ± 0.2 ^a^			60.12 ± 0.6 ^a^			0.1025
Ribeye area (cm^2^)	79.83 ± 0.63 ^a^			78.43 ± 0.45 ^a^			0.0683
Yield grade (points)	2.78 ± 0.06 ^a^			2.96 ± 0.10 ^a^			0.1298
By sex ^5^	Male	Female		Male		Female	
Hot carcass weight (kg)	321.1 ± 3.8 ^a^	317.9 ± 5.2 ^a^		320.0 ± 6.6 ^ab^		296.8 ± 12.3 ^b^	
Dressing percentage (%)	61.07 ± 0.4 ^a^	61.49 ± 0.4 ^a^		60.85 ± 0.4 ^a^		58.73 ± 1.8 ^b^	
Ribeye area (cm^2^)	80.18 ± 0.82 ^a^	79.48 ± 0.98 ^ab^		77.54 ± 0.72 ^ab^		79.43 ± 0.47 ^b^	
Yield grade (points)	2.82 ± 0.08 ^a^	2.73 ± 0.09 ^a^		3.01 ± 0.11 ^a^		2.77 ± 0.21 ^a^	
**Economic analysis**							
ITEM	14%TS			11%TS			*p*-Value/dif.
Average per animal USD	$1569.45 ± 16.1 ^a^			$1484.58 ± 30.2 ^b^			0.0153
Sum USD ^6^	$62,777.99 ± 16.11			$59,383.11 ± 30.20			$3394.88 ^7^
By sex	Male	Female		Male		Female	
Average per animal USD ^8^	$1600.31 ± 18 ^a^	$1538.59 ± 29 ^a^		$1578.01 ± 34 ^a^		$1391.11 ± 40 ^b^	
Sum USD	$32,006.24	$30,771.75		$31,560.97		$27,822.11	
Dif. USD ^9^	+$445.27	+2949.64		−$445.27		−2949.64	

^1^ Lot type represents the grouping of animals based on their growth parameters. Lot type indicates the quality required for the sale of the animals, with type 1 being the highest quality and type 3 the lowest. ^2^ Lot type least squares mean by sex that do not share a common superscript letter (a, b, c) differ significantly (*p* < 0.05) according to Tukey’s HSD test. ^3^ Lot type USD indicates the average sale price per animal in USD for each evaluated lot type. ^4^ Carcass traits least squares mean by group that do not share a common superscript letter differ significantly (*p* < 0.05) according to Tukey’s HSD test. ^5^ Carcass traits least squares mean by group × sex that do not share a common superscript letter differ significantly (*p* < 0.05) according to Tukey’s HSD test. ^6^ SUM USD represents the income obtained in USD for the total number of animals for each group. ^7^ Dif. quantifies the income differential between the groups evaluated. ^8^ Economic analysis least squares mean by group × sex that do not share a common superscript letter differ significantly (*p* < 0.05) according to Tukey’s HSD test. ^9^ Dif. USD represents the income difference between individuals of the same sex and the evaluated groups, indicating either a surplus or a deficit depending on the sign of the value.

**Table 4:** Beef grading and economic analysis of Holstein × beef heifers and steers, based on USDA beef carcass price index and US-approved automatic grading.

**Beef Grading**									
ITEM	14%TS				11%TS				*p*-value
Beef grading (n)	Choice		Select		Choice		Select		
	27		13		20		20		0.1187
By sex (n) ^1^	Male	Female	Male	Female	Male	Female	Male	Female	
	13 ^ab^	14 ^a^	7	6	7 ^b^	13 ^ab^	13	7	
**Economic analysis ^2^**									
ITEM	14%TS				11%TS				*p*-value/dif.
Average per animal USD	$2616.53 ± 26.9 ^a^				$2499.26 ± 39.4 ^b^				0.0155
Sum USD	$104,661.05 ± 26.95				$99,970.36 ± 39.43				$4690.69 ^3^
By sex	Male		Female		Male		Female		
Average per animal USD ^4^	$2630.71 ± 26.5 ^a^		$2602.34 ± 39.9 ^ab^		$2558.88 ± 53.7 ^ab^		$2439.64 ± 56.79 ^b^		
Sum USD	$52,614.17		$52,046.88		$51,177.63		$48,792.73		
Dif. USD	+$1436.54		+3254.15		−$1436.54		−3254.15		
By sex and beef grading	Choice	Select	Choice	Select	Choice	Select	Choice	Select	
Average per animal USD ^5^	$2695.32 ± 41 ^a^	$2510.72 ± 49 ^ab^	$2661.43 ± 40 ^a^	$2464.48 ± 66 ^ab^	$2640.59 ± 78 ^ab^	$2514.88 ± 68 ^ab^	$2479.30 ± 80 ^ab^	$2365.97 ± 63 ^b^	
Sum USD	$35,039.1	$17,575.1	$37,260.0	$14,786.9	$18,484.1	$32,693.5	$32,230.9	$16,561.8	
Dif. USD ^6^	+$16,554.09	−$15,118.45	+$20,698.23	−$17,444.08	−$16,554.09	+$15,118.45	−$20,698.23	+$17,444.08	

^1^ Least squares mean by sex was calculated by group × sex × beef grading (Choice); those that do not share a common superscript letter (a, b) differ significantly (*p* < 0.05) according to Tukey’s HSD test. ^2^ Economic analysis, the cost per animal was estimated for each quality grade as the product of carcass weight (pound) and price per pound, according to the values reported in the USDA Beef Carcass Price Equivalent Index Value (September 2025). ^3^ Dif. quantifies the income differential between the evaluated groups. ^4^ Average per animal USD least squares mean by sex was calculate by group × sex × USD per animal that do not share a common superscript letter differ significantly (*p* < 0.05) according to Tukey’s HSD test. ^5^ By sex and beef grading, least squares mean by sex was calculated by group × sex × beef grading × USD per animal, those that do not share a common superscript letter differ significantly (*p* < 0.05) according to Tukey’s HSD test. ^6^ Dif. USD represents the income difference between individuals of the same sex, same beef grading and the evaluated groups, indicating either a surplus or a deficit depending on the sign of the value.

## Data Availability

The data presented in this study are available on request from the corresponding authors.

## Abbreviations

The following abbreviations are used in this manuscript:

ADG	Average daily gain
BW	Body weight
CP	Crude protein
CW	Carcass weight
CTW	Hundredweight
DM	Dry matter
DP	Dressing percentage
FW	Final weight
LT	Lot type
Mcal	Megacalorie
ME	Metabolizable energy
NDF	Neutral detergent fiber
RA	Ribeye area
SENASICA	National Agro-Alimentary Health, Safety, and Quality Service
TIF	Federal Inspection Type
TMR	Total mixed ration
TS	Total solids
USD	United States Dollar
YG	Yield grades
%CTBRC	Closely trimmed, boneless retail cuts
